# Effect of skeletal muscle loss during neoadjuvant imatinib therapy on clinical outcomes in patients with locally advanced GIST

**DOI:** 10.1186/s12876-022-02479-4

**Published:** 2022-08-26

**Authors:** Ping’an Ding, Honghai Guo, Xiaoxiao He, Chenyu Sun, Scott Lowe, Rachel Bentley, Qin Zhou, Peigang Yang, Yuan Tian, Yang Liu, Li Yang, Qun Zhao

**Affiliations:** 1 The Third Department of Surgery, The Fourth Hospital of Hebei Medical University, Shijiazhuang, 050011 Hebei China; 2 Hebei Key Laboratory of Precision Diagnosis and Comprehensive Treatment of Gastric Cancer, Shijiazhuang, 050011 China; 3 The Third Department of CT/MRI, the Fourth Hospital of Hebei Medical University, Shijiazhuang, 050011 Hebei China; 4 AMITA Health Saint Joseph Hospital Chicago, 2900 N. Lake Shore Drive, Chicago, IL 60657 USA; 5 College of Osteopathic Medicine, Kansas City University, 1750 Independence Ave, Kansas City, MO 64106 USA; 6 Radiation Oncology, Mayo Clinic, 200 First Street SW, Rochester, MN 55905 USA

**Keywords:** Locally advanced gastrointestinal stromal tumors, Skeletal muscle loss, Sarcopenia, Neoadjuvant therapy, Significant muscle loss

## Abstract

**Background:**

Currently, the effect of skeletal muscle loss during neoadjuvant imatinib therapy on clinical outcomes in patients with locally advanced gastrointestinal stromal tumors (LA-GIST) remains unclear. This study aims to investigate the relationship between changes in skeletal muscle and postoperative complications, survival and tumor response in patients with LA-GIST during neoadjuvant therapy with imatinib.

**Methods:**

We retrospectively analyzed pre- and post-treatment computed tomography images of 57 GIST patients who underwent radical surgery after neoadjuvant therapy with imatinib from January 2013 to March 2019. Skeletal muscle index (SMI) was measured at the L3 vertebral level in all patients. A cut-off value (SMI < 52.3 cm^2^/m^2^ and < 38.6 cm^2^/m^2^ for men and women, respectively) published in a previous study was used to define sarcopenia. Based on gender, we defined ΔSMI (%)/250 days above 9.69% for men and ΔSMI (%)/250 days above 7.63% for women as significant muscle loss (SML). Factors associated with postoperative complications and tumor response were analyzed using logistic regression, and predictors affecting patient prognosis were analyzed using Cox regression.

**Results:**

Of the 57 patients, sarcopenia was present before and after neoadjuvant therapy in 20 (35.09%) and 28 (49.12%) patients, respectively. It was not associated with immediate or long-term clinical outcomes. However, patients with SML during neoadjuvant therapy had a higher incidence of postoperative complications (60.00% vs. 25.00%, *p* = 0.008), worse pathological regression (44.00% vs. 75.00%, *p* = 0.017) and worse 3-year survival (Male, 68.75% vs. 95.45%, *p* = 0.027; Female, 66.67% vs. 100.00%, *p* = 0.046) than patients without SML.

**Conclusion:**

The development of SML during neoadjuvant therapy in LA-GIST patients, rather than pre- and post-treatment sarcopenia, is a major prognostic factor for the long-term prognosis and is also associated with recent postoperative complication rates and pathological regression.

## Introduction

Gastrointestinal stromal tumor (GIST) is the most common mesenchymal tissue-derived tumor of the gastrointestinal tract with diverse biological behavior [1, 2]. Previous studies have shown that approximately 95% of GIST patients have mutations in c-kit or platelet derived growth factor receptor alpha (PDGFRA) [3]. Surgery is the treatment of choice for primary GIST, but the 5-year recurrence-free survival rate for patients with locally advanced GIST(LA-GIST) is only 45% to 65% [4, 5]. Meanwhile, Imatinib is a tyrosine kinase inhibitor (TKI), which has been used as a first-line choice for preoperative treatment of LA-GIST patients [6]. Numerous studies have demonstrated that preoperative neoadjuvant therapy with imatinib for LA-GIST significantly improves the rate of complete resection, reduces tumor volume, and decreases the incidence of postoperative complications [7, 8].

Imatinib is usually well tolerated during treatment, but grade 3–4 toxicities have been reported in at least 16% of patients, with up to 40% resulting in discontinuation of the drug [9]. A more common consequence is gastrointestinal toxicity, which leads to weight loss, malabsorption, and even malnutrition [10]. This will result in furter alterations in the patient's skeletal muscle volume during neoadjuvant therapy, and such changes may play an important role in the tumor outcome [11, 12]. Moreover, it is well established that patients with sarcopenia have lower survival rates as well as more toxic chemotherapy, in addition to higher rates of post-operative infection and longer hospital stays after surgery as compared to patients without sarcopenia[13]. Emerging evidence shows that skeletal muscle measurements via CT scans of the L3 vertebrae are thought to be strongly correlated with whole-body skeletal muscle [14, 15]. Numerous studies have confirmed that the area of skeletal muscle and adipose tissue in cross-sectional CT images scanned at the L3 vertebrae level in the supine position correlates closely with whole-body muscle and fat mass [14, 15].

Compared with body mass index (BMI), CT-derived body composition measures assessed at the L3 level better account for gender differences, are able to differentiate between muscle mass and fat mass, and discern the distribution of adipose tissue [16]. A growing number of studies confirm that skeletal muscle results measured at the L3 level are more reflective of the cancer patients’ real status, and their association with clinical outcomes is receiving increasing attention [17, 18]. Currently, abdominal CT examination is widely used for preoperative examination, post-treatment effect evaluation and follow-up of LA-GIST patients [19]. Hence, CT images could be easily used to assess skeletal muscle changes in patients during treatment. Importantly, "opportunistic" skeletal muscle on images acquired for other reasons does not lead to any potential harm or inconvenience for the patient, most inmportantly radiation exposure. Previous studies on the prognosis of LA-GIST have focused on tumor location, size and nuclear fractionation [3, 20]. Nevertheless, the impact of skeletal muscle changes during treatment on patients with LA-GIST remains unclear. Recently, several studies have evaluated the relationship between sarcopenia and the prognosis of GIST patients; however, few studies have analyzed the skeletal muscle changes in LA-GIST before and after neoadjuvant therapy with imatinib, and the impact of these changes on the incidence of recent postoperative complications and long-term prognosis [21, 22].

Therefore, the purpose of this study was to investigate the impact of changes in skeletal muscle before and after neoadjuvant therapy with imatinib on clinical outcomes, as well as to further evaluate whether measurements at the L3 level using conventional CT images could predict the incidence of near-term complications and long-term prognosis of LA-GIST.

## Materials and methods

### Study design and participants

This study retrospectively analyzed 57 patients with LA-GIST who underwent neoadjuvant imatinib therapy in the Fourth Hospital of Hebei Medical University from January 2013 to March 2019. The inclusion criteria were as follows: (1) all patients had histopathologically confirmed GIST; (2) genetic tests suggested the imatinib treatment was indicated; (3) age between 18 and 75 years; (4) preoperative imaging examination showed that the lesions were locally advanced, and surgery without pre-operation chemotherapy or radiation therapy may have a significant impact on the quality of life, including: the tumor site ≤ 5 cm from the cardia; ≤ 5 cm from anal dentate line; ≤ 5 cm from duodenal papilla; pancreaticoduodenectomy or combined organ resection is required for surgery; tumor diameter ≥ 10 cm;(5) all patients were treated with radical surgery after neoadjuvant treatment with imatinib; (6) complete hospitalization data, including CT scans and follow-up data before and after neoadjuvant treatment, were available. The exclusion criteria were as follows: (1) the presence of concurrent tumors other than LA-GIST; (2) the presence of acute bleeding, perforation, and obstruction requiring emergency surgery; (3) poor functional reserve of organs that cannot tolerate surgery or patient refusal to undergo surgical treatment, or patient inability to cooperate with treatment; (4) the presence of lumbar spine metal implants. This study was tested and approved by the ethics committee of the Fourth Hospital of Hebei Medical University. All patients provided informed consent.

### Imatinib neoadjuvant therapy

The decision to administer imatinib neoadjuvant therapy was made by a multidisciplinary panel of surgeons, oncologists, pathologists, and radiologists after all patients were diagnosed with LA-GIST. The initial dose of imatinib was determined based on the results of genetic testing, which resulted in a dose of 400 mg/d for the KIT exon 11 mutation.

Abdominal CT examination was performed every 3 months during preoperative treatment and the efficacy was assessed according to the Choi criteria. According to the National Comprehensive Cancer Network (NCCN) and the Chinese Society of Clinical Oncology (CSCO) guidelines for the treatment of GIST, the recommended duration of pre-operative neoadjuvant imatinib treatment is 6–12 months to maximize the effectiveness of the drug [23, 24]. The optimal timing of surgery was chosen if the either of the two following criteria was met: (1) two consecutive CT scans revealed no regression of the tumor; (2) surgery was considered by the surgeon to be radical and/or organ-preserving. All patients were treated surgically after 1 week of discontinuation of imatinib.

### Measurement and definition of body composition

We retrospectively analyzed CT images before neoadjuvant therapy (1 week before starting oral imatinib) and after neoadjuvant therapy (1 week after stopping oral imatinib). Therefore, in this study, skeletal muscle areas were measured at the L3 vertebrae level in patients with LA-GIST.

All patients were scanned using dual source CT (SIEMENS SOMATOM Definition Flash). The scanning range was from the diaphragm to the iliac crest. The scanning parameters were a voltage of 120 kV, an effective tube current of 300 mA and a slice thickness of 5 mm. 5 mm planar scans were uploaded to the picture archiving and communication system (PACS, SIEMENS SOMATOM) after image acquisition had been completed in all patients. To minimise measurement bias, the trained researcher was not aware of all anthropometric and surgical features. The number of pixels with HU values between [-29, 150] in all segmented regions at the level of the patient's scan was extracted on dedicated processing software (Multimodal Reading software, SIMENS syngo.via) [25] and multiplied by the unit pixel area to obtain the total skeletal muscle cross-sectional area (cm^2^). The software evaluates and measures the pixel area of the area corresponding to the skeletal muscle attenuation to obtain the skeletal muscle area (SMA). For uniform and standardised comparison, the skeletal muscle cross-sectional area was divided by the square of the height to obtain the skeletal muscle index (SMI), and all results are expressed in square metres (cm^2^/m^2^).

**Fig. 1:**
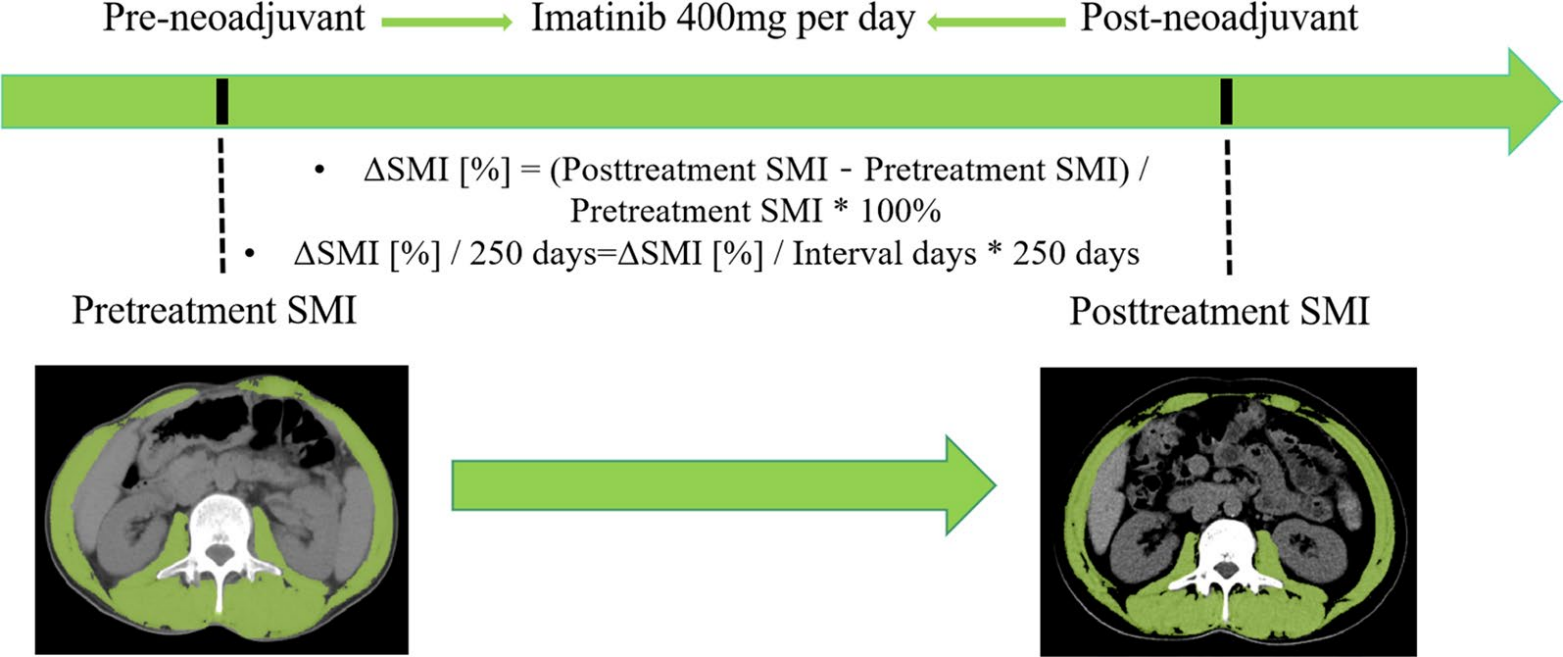
Skeletal muscle measurements at L3 level before and after neoadjuvant therapy

The results of the Martin study were used to define sarcopenia as follows: (1) for patients without obesity (BMI < 30 kg/m^2^), SMI < 52.3 cm^2^/m^2^ and < 38.6 cm^2^/m^2^ were considered as sarcopenia for men and women, respectively; (2) for patients with obesity (BMI ≥ 30 kg/m^2^), SMI < 54.3 cm^2^/m^2^ for men and < 46.6 cm^2^/m^2^ for women were considered as sarcopenia [26]. In addition, considering the different duration of neoadjuvant treatment with imatinib in this cohort of patients, we aimed to provide a standardized unit for comparison between the groups. Therefore, the amount of change in SMI before and after neoadjuvant therapy [ΔSMI] was calculated, while the percentage of this value relative to the pre-neoadjuvant SMI was recorded as ΔSMI[%]. Meanwhile, this value was further divided by the number of days apart and then multiplied by 250 to represent the relative change over 250 days (ΔSMI[%]/250 days) (Fig. 1).

### Outcome measures

The recent observations in this study included the occurrence of postoperative complications and postoperative pathological regression. Postoperative complications were recorded mainly for intestinal obstruction, surgical site infection, healing of the anastomosis, and cardiac and pulmonary related complications. The severity of postoperative complications was also assessed according to the Clavien-Dindo classification validation and score [27].

The criteria for evaluating the pathological efficacy of LA-GIST patients after neoadjuvant therapy were based on the "Chinese consensus guidelines for diagnosis and management of gastrointestinal stromal tumor" published in 2017, which classified postoperative pathological specimens into mild effect (≤ 10%), low effect (> 10% and < 50%), moderate effect (≥ 50% and ≤ 90%), and high effect (> 90%) according to the percentage of necrotic degeneration areas in the tumor tissues [28]. In this study, the mild and low effects were combined into a low response group, and the medium and high effects were combined into a high response group.

The long-term observational endpoint of this study was 3-year survival which was defined as the time interval from the initial date of neoadjuvant therapy with imatinib to the date of last follow-up or death. After initiation of treatment, all patients were followed up every 3 months. In the meanwhile, enhanced CT scans were also be performed every 3 months for the first 3 years after surgery and then every 6 months for the next 2 years. After 5 years, follow-up was performed annually.

### Statistical analysis

SPSS version 21.0 and GraphPad Prism 8.01 were used for statistical analyses. In this study, for continuous variables, normality was tested using the Shapiro–Wilk test, with continuous variables conforming to a normal distribution expressed as mean ± SD, while for non-normally distributed continuous variables, median (interquartile spacing) was used, and statistical tests were compared between groups using independent t-tests or Mann–Whitney U-tests. And categorical data were expressed as numbers and percentages and analyzed using the chi-square test or Fisher's exact test. McNemar's test was used to test for significant differences in paired categorical data. Factors affecting tumour response and postoperative complications were assessed using ordinal logistic regression and all variables with *p* < 0.05 in the univariate analysis were included in the multifactorial analysis. To determine the optimal cut-off value for ΔSMI (%)/250 days, which was used to classify patients into groups with good or poor OS prognosis. The optimal cut-off value for ΔSMI (%)/250 days was obtained using the receiver operating characteristic (ROC) curve for the highest Youden's index. Survival curves were plotted using the Kaplan–Meier method, and the log-rank test was used to compare survival rates between groups. Univariate and multivariate analyses were performed using Cox proportional risk regression models, and results were shown as hazard ratio (HR) with 95% confidence intervals (CI). A *p* < 0.05 was considered statistically significant.

## Results

### Changes in sarcopenia during neoadjuvant therapy

A total of 57 patients with LA-GIST who received preoperative neoadjuvant imatinib and underwent radical surgery were finally enrolled according to the inclusion and exclusion criteria. In this study, no drug withdrawal or dose adjustment was observed in all patients during neoadjuvant therapy. However, four patients had drug-related side effects during oral administration of imatinib, including two patients with facial edema and two patients with bone marrow suppression, which showed a decrease in white blood cells and neutrophils. The 4 patients were relieved after symptomatic treatment, and there was no withdrawal and dose adjustment.

**Fig. 2:**
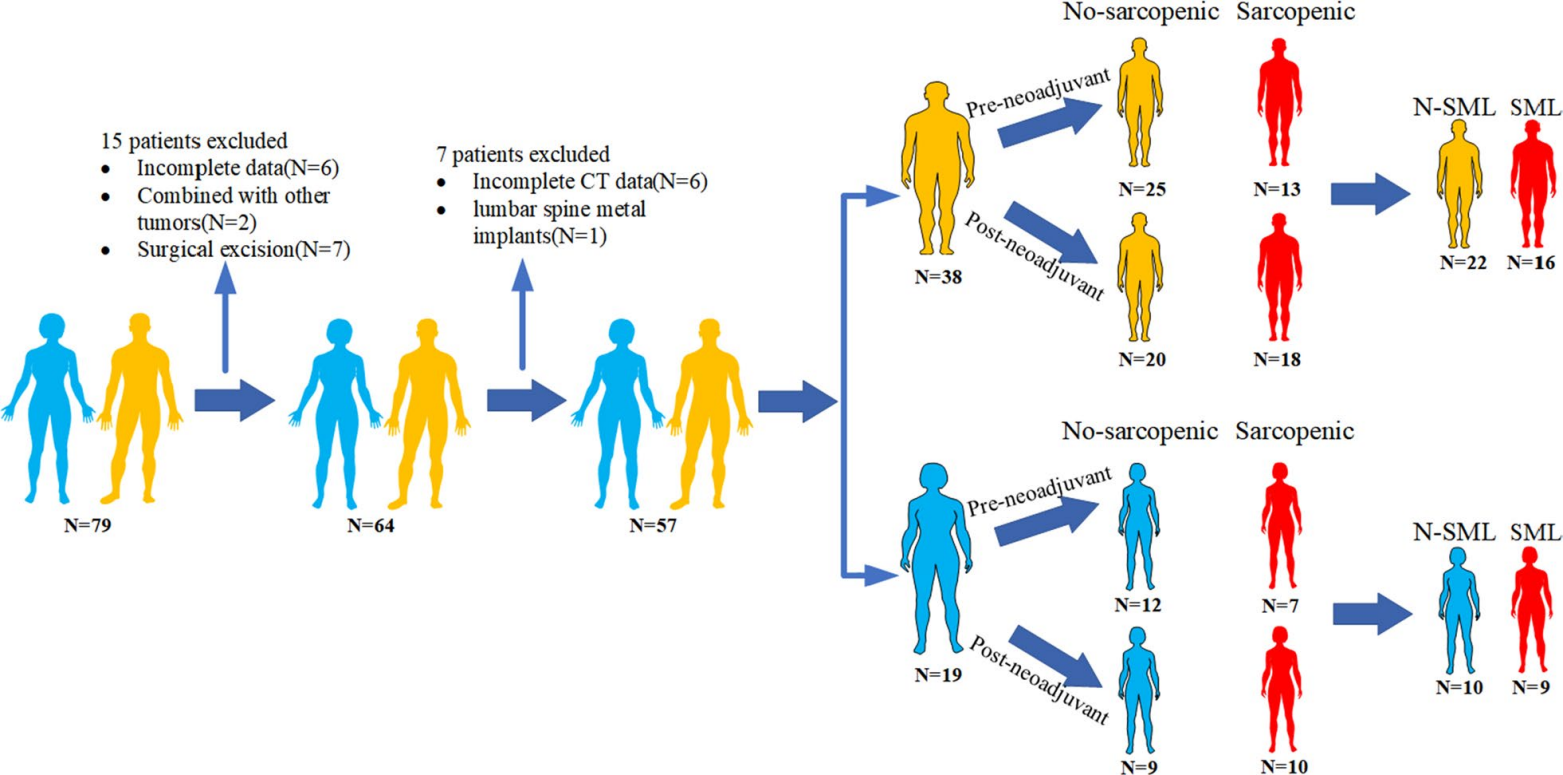
Flow chart of the study. SML: Significant muscle loss. **A**: Male; **B**: Female

**Fig. 3:**
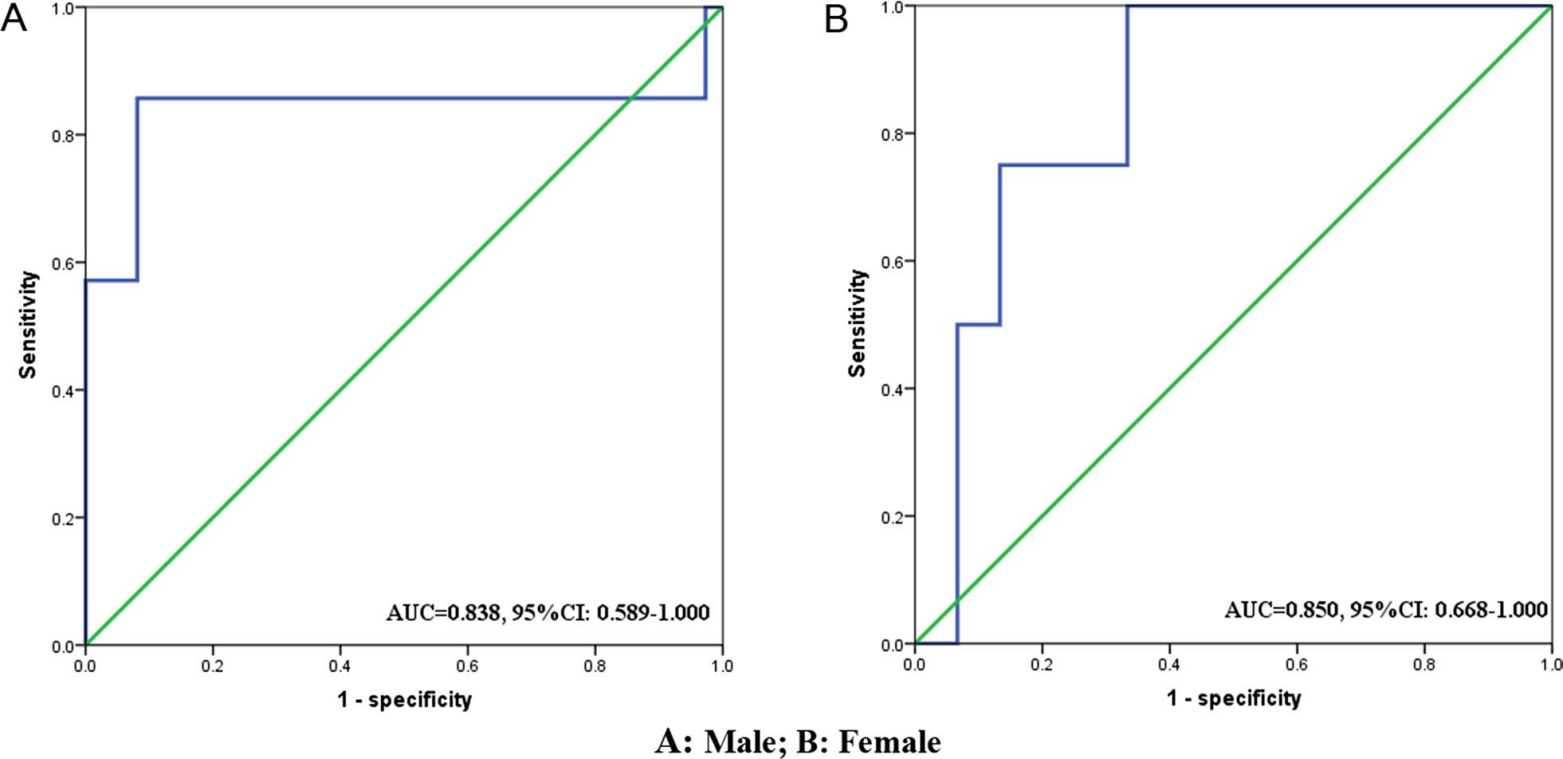
ROC curve to determine the optimal cut-off value for ΔSMI (%)/250 days

Figure 2 shows the detailed flow chart of this study. Twenty (35.1%) LA-GIST patients were already in sarcopenia before neoadjuvant therapy with imatinib, including 13 (65.0%) males and 7 (35.0%) females. After neoadjuvant therapy, 28 (49.1%) patients developed sarcopenia, which was still more common in males (64.3%). The mean ΔSMI (%)/250 days during neoadjuvant therapy was − 7.00 ± 4.73% for the whole group of patients. Since the optimal cut-off value for ΔSMI (%)/250 days has not been clearly defined, we used ROC curves to clarify it. The cut-off value for ΔSMI (%)/250 days in male patients was 9.69% [area under the curve (AUC) = 0.838, 95%CI 0.589–1.000], which corresponded to a sensitivity of 0.833 and a specificity of 0.906, while in female patients the cut-off value was 7.63% [AUC = 0.850, 95%CI 0.668–1.000], which corresponds to a sensitivity of 1.000 and a specificity of 0.588 (Fig. 3). Therefore, 25 (43.9%) patients were classified in the significant muscle loss (SML) group, while 16 (64.0%) of them were male, still a higher percentage than female. Meanwhile, the average duration of patients in the SML group was 267.5 days, while that in the No-SML group was 272.1, and the difference was not statistically significant (*p* = 0.120).

### Patient characteristics

The mean age of the whole group of patients was 57.4 ± 10.7 years, of which 38(66.67%) cases were male. The clinicopathological features and baseline laboratory findings of all patients are shown in Table 1. Although there was no significant difference in baseline tumor characteristics between the two groups before and after neoadjuvant therapy, albumin (− 3.12 ± 4.13 vs. − 0.79 ± 7.02, *p* = 0.036) as well as hemoglobin (− 0.93 ± 3.33 vs. − 0.33 ± 1.03, *p* = 0.043) levels were significantly lower in patients who developed SML during neoadjuvant therapy than in patients without significant muscle loss (No-SML). Furthermore, the decrease in BMI during neoadjuvant was greater in the SML group than in the No-SML group (− 1.02 ± 0.66 vs. − 0.62 ± 0.6, *p* = 0.021).

**Table 1:** Patient and tumor characteristics (N = 57)

Characteristic	Overall (n = 57)	Pre-neoadjuvant		*p*	Post-neoadjuvant		*p*	ΔSMI (%)/250 days		*p*
		Sarcopenia (n = 20)	No-sarcopenia (n = 37)		Sarcopenia (n = 30)	No-sarcopenia (n = 27)		SML (n = 25)	No-SML (n = 32)	
Age (years), mean ± SD	57.4 ± 10.7	56.0 ± 11.5	58.1 ± 10.4	0.723	57.6 ± 11.3	57.1 ± 10.2	0.885	57.9 ± 10.0	56.9 ± 11.4	0.735
*ECOG, n (%)*										
0	49 (86.00%)	15 (75.00%)	34 (91.89%)	0.080	24 (80.00%)	25 (92.59%)	0.172	17 (68.00%)	32 (100.00%)	0.002
1	8 (14.00%)	5 (15.00%)	3 (8.11%)		6 (20.00%)	2 (7.41%)		8 (32.00%)	0 (0)	
*BMI (Kg/m*^*2*^*), mean* ± *SD*										
Pre-neoadjuvant	22.82 ± 3.45	21.87 ± 3.32	23.33 ± 3.45	0.128	22.72 ± 3.67	22.93 ± 3.25	0.827	22.00 ± 3.26	23.46 ± 3.51	0.112
Post-neoadjuvant	22.02 ± 3.49	20.40 ± 3.40	22.85 ± 3.54	0.338	21.80 ± 3.28	22.21 ± 3.71	0.665	20.95 ± 3.15	22.85 ± 3.56	0.040
Δneoadjuvant	− 0.79 ± 0.67	− 0.98 ± 0.66	− 0.45 ± 0.55	0.003	− 1.11 ± 0.62	− 0.51 ± 0.59	0.000	− 1.02 ± 0.66	− 0.62 ± 0.64	0.021
*Gender, n(%)*										
Male	38 (66.67%)	13 (65.00%)	25 (67.57%)	0.844	20 (66.67%)	18 (66.67%)	1.000	16 (64.00%)	22 (68.75%)	0.706
Female	19 (33.33%)	7 (35.00%)	12 (32.43%)		10 (33.33%)	9 (33.33%)		9 (36.00%)	10 (31.25%)	
*Hemoglobin* (*g/L), mean* ± *SD*										
Pre-neoadjuvant	118.86 ± 14.95	117.02 ± 15.24	122.26 ± 14.15	0.210	115.83 ± 14.00	121.59 ± 15.48	0.148	115.80 ± 16.03	122.77 ± 12.72	0.081
Post-neoadjuvant	116.58 ± 13.32	114.89 ± 12.75	119.70 ± 14.10	0.196	112.75 ± 12.41	120.03 ± 13.36	0.038	112.01 ± 13.70	121.32 ± 12.32	0.320
Δneoadjuvant	− 2.27 ± 6.13	− 2.56 ± 4.13	− 2.13 ± 7.02	0.805	− 3.08 ± 5.09	− 1.56 ± 6.94	0.355	− 3.12 ± 4.13	− 0.79 ± 7.02	0.036
*Albumin* (*g/L), mean* ± *SD*										
Pre-neoadjuvant	40.27 ± 3.42	37.95 ± 3.54	41.52 ± 2.64	0.000	39.08 ± 3.67	41.59 ± 2.59	0.005	39.14 ± 4.15	41.16 ± 2.44	0.026
Post-neoadjuvant	38.57 ± 3.01	35.05 ± 2.95	40.39 ± 2.74	0.004	37.46 ± 2.74	39.80 ± 2.86	0.003	38.21 ± 3.82	40.84 ± 2.22	0.432
Δneoadjuvant	− 1.70 ± 2.57	− 2.14 ± 2.37	− 1.24 ± 2.17	0.012	− 1.62 ± 2.66	− 1.79 ± 2.50	0.806	− 0.93 ± 3.33	− 0.33 ± 1.03	0.043
*Tumor site, n (%)*										
Stomach	49 (86.00%)	18 (90.00%)	31 (83.78%)	0.519	28 (93.33%)	21 (77.78%)	0.091	21 (84.00%)	28 (87.50%)	0.706
Non-stomach	8 (14.00%)	2 (10.00%)	6 (16.22%)		2 (6.67%)	6 (22.22%)		4 (16.00%)	4 (12.50%)	
Tumor size (cm), mean ± SD	11.45 ± 4.41	11.29 ± 3.16	11.54 ± 5.00	0.838	11.12 ± 3.56	11.81 ± 5.24	0.557	11.33 ± 3.70	11.54 ± 4.95	0.861

*ECOG* Eastern Cooperative Oncology Group, *BMI* body mass index, *SML* Significant muscle loss

### Association of sarcopenia with immediate postoperative outcomes

**Fig. 4:**
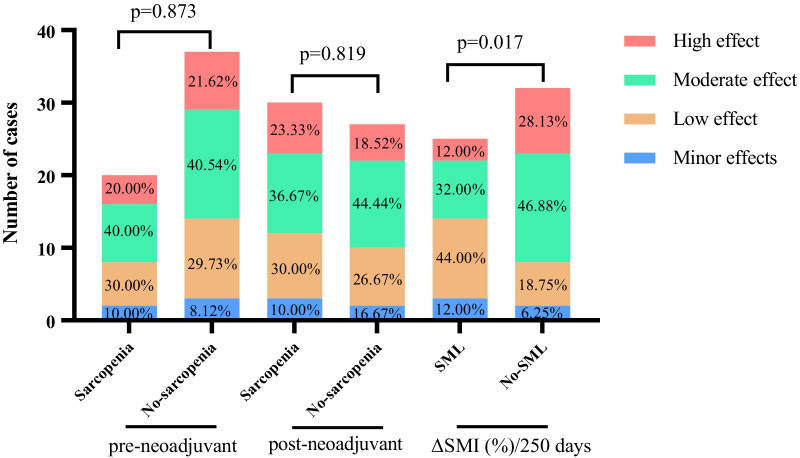
Postoperative pathological regression response after neoadjuvant therapy in patients with LA-GIST

The postoperative pathological response was mild in 5 patients (8.77%) and low 17 (29.82%) patients, while moderate and high effect in 23 (40.35%) and 12 (21.05%) patients, respectively. The rate of high pathological reaction was non-statistically significant lower in both pre- (60.00% vs. 62.16%, *p* = 0.873) and post-neoadjuvant (60.00% vs. 62.96%, *p* = 0.819) sarcopenic group than in the non-sarcopenic group, whereas, a statistically significantly lower rate of high pathological reaction (44.00% vs. 75.01%, *p* = 0.017) in the SML group compared with No-SML group was observed. The above results are shown in Fig. 4.

Meanwhile, all 57 LA-GIST patients underwent radical surgical resection, and 23 cases (40.35%) developed postoperative complications. Most complications were Clavien-Dindo grade I-II, and no fatal cases occurred. There was no significant difference in the incidence of postoperative complications between the sarcopenic and non-sarcopenic groups before neoadjuvant therapy (45.00% vs. 37.84%, *p* = 0.599) and between the sarcopenic and non-sarcopenic groups after neoadjuvant therapy (46.67% vs. 33.33%, *p* = 0.306). In contrast, the SML group had a significantly higher rate of postoperative complications than the No-SML group (60.00% vs. 25.00%, *p* = 0.008) and a higher rate of Clavien-Dindo grade III than the No-SML group (16.00% vs. 0%, *p* = 0.257) (Table 2).

**Table 2:** Correlation between skeletal muscle status and postoperative complications (N = 57)

Variable	Pre-neoadjuvant		*p*	Post-neoadjuvant		*p*	ΔSMI (%)/250 days		*p*
	Sarcopenia (n = 20)	No-sarcopenia (n = 37)		Sarcopenia (n = 30)	No-sarcopenia (n = 27)		SML (n = 25)	No-SML (n = 32)	
Clavien-Dindo classification			0.517^b^			0.517^b^			0.154^b^
I ~ II	7 (35.00%)	12 (32.43%)		12 (40.00%)	7 (25.93%)		11 (44.00%)	8 (25.00%)	
III	2 (15.00%)	2 (5.41%)		2 (6.67%)	2 (7.41%)		4 (16.00%)	0 (0)	
Total^a^	9 (45.00%)	14 (37.84%)	0.599	14 (46.67%)	9 (33.33%)	0.306	15 (60.00%)	8 (25.00%)	0.008
Wound infection	2 (10.00%)	1 (2.70%)	0.279	2 (6.67%)	1 (3.70%)	0.617	3 (12.00%)	0 (0)	0.079^b^
Anastomotic leakage	3 (15.00%)	2 (5.41%)	0.332	4 (13.33%)	1 (3.70%)	0.199	4 (16.00%)	1 (3.13%)	0.157
Lymphatic leakage	1 (5.00%)	2 (5.41%)	0.948	2 (6.67%)	1 (3.70%)	0.617	3 (12.00%)	0 (0)	0.079^b^
Abdominal infection	2 (10.00%)	2 (5.41%)	0.607	3 (10.00%)	1 (3.70%)	0.353	3 (12.00%)	1 (3.13%)	0.309
Abdominal bleeding	1 (5.00%)	3 (8.12%)	0.661	2 (6.67%)	2 (7.41%)	0.913	3 (12.00%)	1 (3.13%)	0.309
Anastomotic bleeding	1 (5.00%)	2 (5.41%)	0.948	2 (6.67%)	1 (3.70%)	0.617	3 (12.00%)	0 (0)	0.079^b^
Intestinal obstruction	2 (10.00%)	1 (2.70%)	0.239	2 (6.67%)	1 (3.70%)	0.617	3 (12.00%)	0 (0)	0.079^b^
Respiratory complications	5 (25.00%)	3 (8.12%)	0.114	6 (20.00%)	2 (7.41%)	0.172	5 (20.00%)	3 (9.38%)	0.016
Cardiovascular complications	3 (15.00%)	2 (5.41%)	0.332	4 (13.33%)	1 (3.70%)	0.199	3 (12.00%)	2 (6.25%)	0.645

*SMI* skeletal muscle index, *SML* Significant muscle loss

^a^Since multiple complications may occur simultaneously in the same patient, the sum of each sub-item is not equal to that of the parent

^b^Calculated by Fisher’s exact test

### The relationship between sarcopenia and long-term prognosis after surgery

**Fig. 5:**
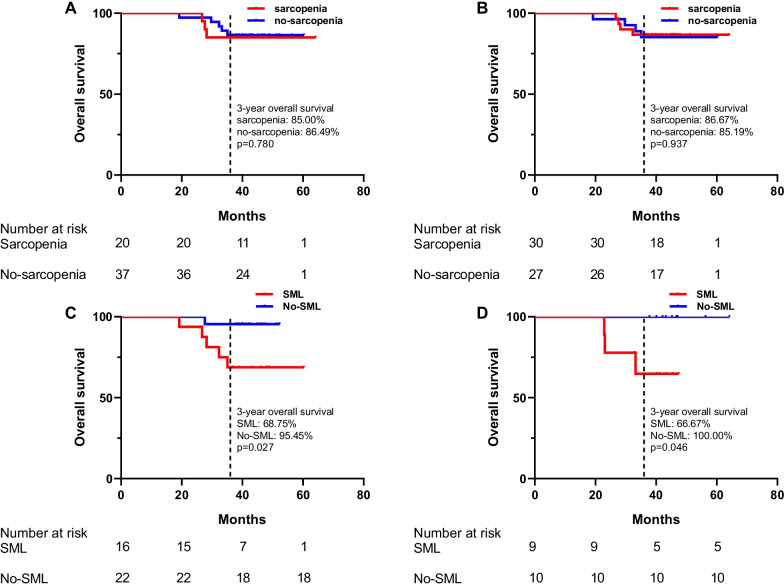
**A** 3-year survival of pre-treatment sarcopenia versus pre-treatment non-sarcopenia; **B** 3-year survival between post-treatment sarcopenia and post-treatment non-sarcopenia; **C** 3-year survival between the appearance of SML and No-SML during neoadjuvant therapy in men. **D** 3-year survival between the appearance of SML and No-SML during neoadjuvant therapy in women

The follow-up time ranged from 19.2 to 64.2 months, with a median follow-up time of 42.1 months. There were 8 deaths (14.04%) and the 3-year survival rate for the entire group was 85.96%. According to log-rank test, there was no significant difference in 3-year survival between the pre-neoadjuvant sarcopenia and non-sarcopenia groups (85.00% vs. 86.49%; *p* = 0.827, Fig. 5A) and between the post-neoadjuvant sarcopenia and non-sarcopenia groups (86.67% vs. 85.19%, *p* = 0.906, Fig. 5B). Nevertheless, a further subgroup analysis based on gender revealed that the 3-year survival rates were significantly lower in the SML group than in the No-SML group (Male, 68.75% vs. 95.45%, *p* = 0.027; Female, 66.67% vs. 100.00%, *p* = 0.046, Fig. 5C-D).

Further Cox regression analysis was performed to analyze the risk factors affecting 3-year survival in patients with LA-GIST. Univariate analysis showed that the presence of SML during neoadjuvant therapy, tumor diameter, mitotic index, baseline albumin, and whether to continue imatinib therapy postoperatively were significantly associated with 3-year survival (Table 3). Meanwhile, neither pre-neoadjuvant sarcopenia (HR = 1.173, 95%CI 0.271–5.072) nor post-neoadjuvant sarcopenia (HR = 1.087, 95%CI 0.272–4.351) was risk factors for poor prognosis, which is the same as the results in Fig. 5. Subsequent multivariate analysis showed that the presence of SML during neoadjuvant remained an independent risk factor for OS (HR = 6.922, 95%CI 2.892–16.091). In addition, whether to continue imatinib treatment after surgery (HR = 0.018, 95% CI 0.005–0.376,) were also significant prognostic factors for OS (Table 3).

**Table 3:** Univariable and multivariable analysis for overall survival (N = 57)

Variable	Univariable analysis	Multivariable analysis
	HR (95% CI)	HR (95% CI)
*Age (years)*		
≤ 60	Reference	
> 60	0.179(0.044–0.720)	
*Gender*		
Female	Reference	
Male	0.630 (0.147–2.705)	
*ECOG*		
1	Reference	
0	0.432 (0.053–3.489)	
*Pre-neoadjuvant BMI (Kg/m*^*2*^*)*		
Underweight (< 18.5)	Reference	
Normal (≥ 18.5)	0.341 (0.013–9.176)	
*Post-neoadjuvant BMI (Kg/m*^*2*^*)*		
Underweight (< 18.5)	Reference	Reference
Normal (≥ 18.5)	0.065 (0.007–0.599)	0.152 (0.005–4.462)
*Pre-neoadjuvant sarcopenia*		
No	Reference	
Yes	1.173 (0.271–5.072)	
*Post-neoadjuvant sarcopenia*		
No	Reference	
Yes	1.087 (0.272–4.351)	
*ΔSMI (%)/250 days*		
No-SML	Reference	Reference
SML	9.958 (2.437–40.680)	6.922 (2.892–16.091)
*Pre-albumin (g/L)*		
≥ 40	Reference	
< 40	2.610 (0.630–10.810)	
*Post-albumin (g/L)*		
≥ 40	Reference	
< 40	1.419 (0.348–5.798)	
*Tumor size (cm)*		
5–10	Reference	
> 10	1.037 (0.259–4.419)	
*Tumor site*		
Stomach	Reference	
Non-stomach	0.461 (0.060–3.544)	
*Postoperative imatinib treatment*		
No	Reference	Reference
Yes	0.013 (0.001–0.128)	0.018 (0.005–0.376)

*ECOG* Eastern Cooperative Oncology Group, *BMI*: body mass index, *SML* Significant muscle loss

## Discussion

This study investigated the impact of sarcopenia and skeletal muscle loss during neoadjuvant therapy with imatinib in patients with LA-GIST on complications, tumor response, and survival outcomes after undergoing radical surgery. Firstly, we observed sarcopenia in about 1/3 of LA-GIST patients before and after neoadjuvant therapy, and 43.86% of patients developed SML during neoadjuvant therapy. Secondly, we found a strong correlation between laboratory indicators related to nutritional status and sarcopenia. Thirdly, based on biological sex, we defined ΔSMI (%)/250 days above 9.69% for men and ΔSMI (%)/250 days above 7.63% for women as significant muscle loss (SML). Meanwhile, we found that the presence of SML during neoadjuvant therapy was significantly associated with a high degree of pathologic translation and the occurrence of postoperative complications in patients.

In previous studies, the long-term prognosis of underweight patients with malignancies (BMI < 18.5 kg/m^2^) was found to be worse than patients who are obese, overweight or those with normal weight, hence BMI has often been used to predict patient prognosis in some studies [29, 30]. Nevertheless, some studies have found that it is not an effective predictor of prognosis, as BMI does not take into account for sex differences and proportions of muscle and adipose tissue, resulting in substantial differences in the ratio of muscle to fat mass between patients with the same BMI [16, 17].Over the past decades, body composition analysis based on CT measurements has shown increasing popularities in clinical practices, with skeletal muscle assessment at the L3 level being the most prevalent [14, 15]. Currently, abdominal CT examinations are widely utilized to evaluate the size of lesions, assessment of efficacy, and follow-up of patients with LA-GIST [28]. As such, all patients in this study underwent abdominal CT measurements before and after neoadjuvant therapy with imatinib. Furthermore, CT images can provide objective measurements of skeletal muscle and fat, especially when assessed dynamically before and after neoadjuvant therapy to better reflect the changes in body composition. In this study, the time point of measurement was chosen before and after neoadjuvant therapy, which helped the patient to avoid the additional radiation exposure.

To the best of knowledge, there is no consensus on the optimal cut-off values for sarcopenia in patients with malignancy and for skeletal muscle changes during neoadjuvant therapy. In this study, we defined sarcopenia based on the results of a previous study by Martin et al. [26]. However, for the optimal cut-off values of skeletal muscle changes during neoadjuvant therapy, we chose to define it differently based on biological sex using ROC curves. In addition, we found that 35.09% of patients developed sarcopenia before neoadjuvant therapy, which is consistent with the results of previous studies [21, 31]. In a previous retrospective analysis of 248 male patients diagnosed with esophageal squamous cell carcinoma and treated with neoadjuvant radiotherapy followed by surgery, 70 (28.2%) patients showed excessive muscle loss [32]. As for LA-GIST, our results found significant muscle loss in 43.86% of patients. Consequently, these results suggest that, as with other gastrointestinal malignancies, LA-GIST patients also suffer from significant muscle loss in neoadjuvant therapy.

A recent study of 1630 patients who underwent surgical resection for colon cancer showed that low preoperative SMI was associated with higher rates of postoperative complications [33]. A study published by Song et al. found that low SMI in skeletal muscle measured at the L3 level on preoperative CT scans may be a promising predictor of postoperative complications in patients with GIST [21]. However, our study found that the presence of low SMI before or after neoadjuvant therapy did not correlate with the incidence of postoperative complications in patients with LA-GIST. Interestingly, we found that significant skeletal muscle loss during neoadjuvant therapy in LA-GIST patients was strongly associated with the occurrence of postoperative complications and with Clavien-Dindo classification. In addition, a retrospective study that included 31 patients with esophageal cancer who underwent surgery after neoadjuvant chemotherapy found that a better pathological regression response was more common in the non-muscular reduction group than in the muscular reduction group (53.3% vs. 25.0%) [34]. In regards to GIST, our study revealed that the presence of sarcopenia before and after neoadjuvant therapy was not associated with a pathologic regression response, while patients who developed significant muscle loss during treatment had a significantly worse pathologic response than patients who did not develop significant muscle loss. In essence, the mechanism of the association between significant muscle loss and low pathological withdrawal rate is unclear, so prospective studies with large sample sizes are needed in the future.

Currently, most studies on the prognostic impact of sarcopenia have focused on cancer patients, while few studies have focused on the prognostic impact of sarcopenia on GIST patients, especially for LA-GIST patients on neoadjuvant therapy [3–8]. Until now, only one study has investigated the impact of sarcopenia on the prognosis of patients with GIST [21]. This study included 107 patients who underwent surgical resection, which found that patients with sarcopenia had a significantly lower median OS (40.6 months; 95% CI 31.6–49.6) than patients with normal skeletal muscle (63.8 months; 95% CI 57.6–69.8; *p* = 0.0011). In our study, there was no significant difference in the prognosis of patients with and without sarcopenia before and after neoadjuvant therapy. However, further analysis of the prognostic impact of the amount of muscle loss during neoadjuvant therapy revealed that patients who developed significant muscle loss suffered a significantly worse prognosis than the normal group.

At present, it is difficult to derive a causal relationship between significant muscle loss during neoadjuvant therapy and OS. However, recent studies have discovered that skeletal muscle produces and releases a cytokine that is further involved in the anti-inflammatory response, including mediating the interaction of the pro-inflammatory cytokine interleukin-6 with NK cells to induce the production of IL-1 receptor antagonists and IL-10, thereby acting as an anti-tumour agent [35, 36]. This suggests that skeletal muscle status affects tumourigenesis and systemic inflammation,and conversely, significant muscle loss during neoadjuvant therapy could be associated with altered inflammatory response and impaired anti-tumour immunity [37]. Furthermore, considering that LA-GIST patients on neoadjuvant therapy experience dysphagia and anorexia as a result of drug reactions, malnutrition might occur, which can further exacerbate skeletal muscle loss. In spite of the positive impact of imatinib neoadjuvant therapy on LA-GIST patients survival, significant muscle loss during treatment will adversely affect both the immediate clinical outcome and the long-term prognosis of patients. Therefore, we also need to pay more attention to the nutritional status of LA-GIST patients during neoadjuvant therapy in order to be able to improve their prognosis.

Certain limitations of this study merit consideration. First, this study was a single-center retrospective study, and there may be some selection bias in the study population. Second, this study only explored the effects of skeletal muscle changes on postoperative complications, pathological regression reactions, and prognosis at two time points. Third, the subjects included in this study had low BMI, resulting in a small dose of imatinib. We did not investigate the adverse reactions caused by different doses of oral imatinib during neoadjuvant therapy. Adverse drug reactions, especially the presence of gastrointestinal reactions can affect the patient's nutrition and further affect muscle loss. Therefore, the association between sarcopenia and adverse drug reactions during neoadjuvant therapy need to be further analyzed in the follow-up prospective study. Meanwhile, subgroup analysis can also be further carried out according to the different BMI of the study population. Finally, given its retrospective design, we were unable to determine a causal relationship between sarcopenia and poor prognosis. In addition, due to the small number of patients included in this study, the cut-off value of skeletal muscle loss derived from the ROC curve is prone to overfitting to the data set used, thereby biasing its accuracy. Therefore, in future studies, we will further expand the sample size and carry out multicenter, prospective studies to further verify this result.

## Conclusions

In conclusion, in this study, skeletal muscle measurements at L3 vertebrate level based on abdominal CT scans could be used to predict the immediate and long-term clinical outcomes of LA-GIST patients after neoadjuvant therapy. More importantly, significant muscle loss during neoadjuvant therapy rather than sarcopenia before and after neoadjuvant therapy is a risk factor for patient prognosis.

## Data Availability

The datasets used and/or analyzed during the current study are available from the corresponding author on reasonable request.
